# Bromide of Ethyl the Most Perfect Anæsthetic for Short Operations

**Published:** 1883-09

**Authors:** J. J. Chisolm


ARTICLE V.

### BROMIDE OF ETHYL THE MOST PERFECT ANÆSTHETIC FOR SHORT OPERATIONS.

Dr. J. J. Chisholm, Professor of Eye and Ear Diseases in the University of Maryland, Surgeon in Charge of the Presbyterian Eye and Ear Charity Ho pital, Ophthalmic Surgeon to the University Hospital, etc., of Baltimore, says: The recumbent position I consider essential for the safe administration of any anæsthetic, whether it be chloroform, ether or ethyl, hence these agents are not safe remedies at the hands of dentists, who place their patients in a sitting posture. Preparatory to the inhalation of the bromide of ethyl I have not found it necessary to give whiskey. The only precaution I take is to loose the neck clothing and have the patient lie down with the head only slightly elevated.

*My experiments have taught me that the mode of administering the ethyl should differ totally from that used in giving chloroform.*

Instead of a chloroform vapor freely diluted with atmospheric air, a saturated ethyl vapor must be inhaled, to the exclusion of atmospheric air, in order to obtain speedily and effectually narcosis.

In my early experiments with this new agent I had not yet discovered this fundamental principle, and hence did not obtain good results. I voted bromide of ethyl a failure, because in common with other experimenters, I was too timid, or rather I should say too ignorant of its peculiarities, to push the ethyl vapor in the concentrated form, which I have since found necessary to obtain good results. By my present method of administering it, I can obtain perfect ethylization in patients in from twenty to sixty seconds, and have no after consequences of nausea or dullness of feeling.

The best inhaler for the giving of the bromide of ethyl is a thick towel folded into the form of a small cone with closed apex. Between one of the folds of the towel I place a sheet of paper, which makes the cone nearly air-tight. The base of the cone must be wide enough to enclose both mouth and nose. The soft material of which the inhaler is made enables the rim to be kept firmly in contact with the face, so as to exclude air from entering. I always instruct the patient how to make long inspirations, and inform him that he must do this, notwithstanding the fact that he will feel somewhat stifled. I also try to give hlm confidence by assuring him that a very few inspirations will put him to sleep. Usually I make him go through the process of strong respiratory movements in advance, so that he will know exactly how to proceed. Into this towel cone I pour about one drachm of the bromide of ethyl and immediately invert the inhaler over the nose and mouth of the patient, holding its edge down firmly over the face. There is no fear of creating asphyxia, as all air cannot be excluded, and the hight of the cone makes a considerable air chamber into which the patient breathes.

Children usually struggle to escape from the apparatus. *The cone, however, must not be removed from the face for an instant until anæsthesia is produced.* At first some patients will resist the breathing of the vapor, but there is no fear that they will not catch their breath in time. Should children cry, it only insures respiratory efforts, which the more surely and quickly will bring about the introduction of the vapor into the lungs. As a rule, a dozen full inspirations are all that are needed to produce deep narcosis. I recognize this desirable condition by a stoppage of all struggling. I have had deep sleep brought on by the sixth inspiration, when complete relaxation ensues, with quiet breathing, and an absence of reflex irritation should the conjunctive be touched. The patient retains the usual healthy color of lips and cheeks as if in ordinary sleep, and the pulse becomes slower and stronger as the narcosis becomes profound. Thirty seconds, as a rule, is sufficient to bring about this desirable condition, and have the patient ready for operation.

I have not found this anæsthetic sleep last more than two or three minutes, often not so long.

Usually the patients awake suddenly and as completely as they would do from ordinary sleep. They are able to get down from the operating table without assistanee and walk off without staggering, and with brain clear to answer correctly any question: in fact, quite themselves.

It took me some time to acquire such confidence in the safety of the remedy, as to apply it in the concentrated form needful to obtain its fullest benefits. To the uninitiated it looks like cruel work to keep the cone of a saturated ethylized vapor over the face of a struggling patient. *I am convinced, however; that in no other way can quick, complete and safe anæsthesia be obtained by it.* Fortunately the struggling is very soon over, and quiet sleep speedily ensues.

My experience with the bromide of ethyl will now exceed 400 cases, of which upwards of 300 are within the past year. I am beginning to be familar with its administration and its effects. *I now know what is to be obtained by it, and what not to expect from it.* I give it without hesitation, in any case, to avoid painful manipulation. I have used it as often as six times a day, and I administer it on an average, certainly once every day. In the last week I have given it fifteen times. For office use I find it invaluable, on account of its promptness, efficiency, evanescent nature of the anæsthesia induced, the absence of nausea, and the perfect comfort with which patients operated upon can leave my office within a few minutes after the ethylization. Its use in my every-day experience does not interfere with the routine of office practice, nor occupy more time than I give to an ordinary office consultation, a very important desideratum to those who have restless patients awaiting their turn in the reception room.

Those who will use it by a single inhalation, to produce a short, deep sleep, and not resort to a mal-administration of this very valuable, powerful agent for a continued anæsthesia, which is incapable of sustaining in safety and in comfort, will become as enthusiastic as I am over its brilliant results. They will in time learn to consider it, as I do, the most perfect of anæsthetic agents for quick, painful surgical work. It can never take the place of chloroform or sulphuric ether where any heavy operations are to be done. These well-known and tried anæsthetics must continue in favor for all tedious operations, and will be used in minor surgery by those who manipulate slowly and who do not have prompt quick assistants. But when one can take advantage of a primary anæsthesia from the first administration of the bromide of ethyl, and having made every preparation in advance, will manipulate quickly, the new anæsthetic leaves nothing to be desired.

I will repeat, "can anything be more brilliant in surgery than a successful operation for squint, where an ugly deformity of years standing is promptly, thoroughly, safely and surely removed in less than one minute of time—fifty-two seconds for ethylization and operation?" This is the nearest approach to magic in the art of surgery.—*Maryland Medical Journal.*

